# The Expression and Function of Circadian Rhythm Genes in Hepatocellular Carcinoma

**DOI:** 10.1155/2021/4044606

**Published:** 2021-10-16

**Authors:** Yanan Jiang, Xiuyun Shen, Moyondafoluwa Blessing Fasae, Fengnan Zhi, Lu Chai, Yue Ou, Hai Feng, Siwei Liu, Ying Liu, Shucai Yang

**Affiliations:** ^1^Department of Pharmacology (State-Province Key Laboratories of Biomedicine-Pharmaceutics of China, Key Laboratory of Cardiovascular Research, Ministry of Education), College of Pharmacy, Harbin Medical University, Harbin, China; ^2^Translational Medicine Research and Cooperation Center of Northern China, Heilongjiang Academy of Medical Sciences, Harbin, China; ^3^Department of Pharmacy, Inner Mongolia Cancer Hospital, Hohhot, China; ^4^Department of Anatomy, Harbin Medical University, Harbin, China; ^5^Department of Nutrition and Food Hygiene, Public Health College, Harbin Medical University, Harbin, China

## Abstract

Hepatocellular carcinoma (HCC) is among the most common and lethal form of cancer worldwide. However, its diagnosis and treatment are still dissatisfactory, due to limitations in the understanding of its pathogenic mechanism. Therefore, it is important to elucidate the molecular mechanisms and identify novel therapeutic targets for HCC. Circadian rhythm-related genes control a variety of biological processes. These genes play pivotal roles in the initiation and progression of HCC and are potential diagnostic markers and therapeutic targets. This review gives an update on the research progress of circadian rhythms, their effects on the initiation, progression, and prognosis of HCC, in a bid to provide new insights for the research and treatment of HCC.

## 1. Introduction

Liver cancer, one of the commonest malignancies, accounts for 4.7 percent of new cancer cases (841,080 new cases) and 8.2 percent of cancer deaths (782,000 deaths) in 2018 globally [1]. Hepatocellular carcinoma (HCC) is the prominent form of primary hepatic cancer, comprising 75%-85% cases of primary liver cancer [1]. HCC is characteristically silent with slow growth and rarely detected at the initiation stage, and it is often clinically diagnosed at an advanced stage or discovered during diagnosis of other related diseases.

Surgery and chemotherapy are the conventional forms of HCC treatment. Surgical resection by either open or laparoscopic surgery is the first choice for most HCC patients. After surgery, most HCC patients still need to undergo chemotherapy [2]. Unfortunately, over 70% of diagnosed HCC cases are already at the late stage, which is not amenable to curative treatment. Especially in the late stages, chemotherapy is the only choice for HCC patients. Till date, drug resistance from cancer cells and side effects of anticancer drugs are major challenges of chemotherapeutic treatment on HCC. Even target-specific drugs, which are considered as the most advanced and effective drug, could only prolong a patient's lifespan for several months. Due to poor diagnosis and prognosis, HCC therapy is still a conundrum for the medical field.

Circadian rhythms are fundamental biological systems in most organisms. Circadian rhythms have been extensively investigated in different species [3–5]. Over the years, the function of circadian genes has aroused the attention of researchers. In 2017, three American geneticists shared the Nobel Physiology or Medicine Prize for their extensive research in the discovery of mechanistic principles involved in the control of circadian rhythms. Various epidemiological and experimental findings have demonstrated that disruption of circadian rhythms is associated with mammalian tumorigenesis and progression [6]. The present paper reviewed the recent understanding on the role of circadian rhythms in HCC, in order to provide new insights in the research and treatment of HCC.

## 2. Circadian Rhythms

Mammalian circadian rhythms arise from the master clock situated in the suprachiasmatic nucleus (SCN), which drives the peripheral clock and synchronizes with environmental signal through circadian input path [7, 8]. Circadian rhythms could generate oscillatory behaviour independent of external factors [9–11]. Currently, at least 15 core circadian genes have been identified, namely: *PER1*, *PER2*, *PER3*, *CLOCK*, *CRY1*, *CRY2*, *ARNTL*/*BMAL1*, *TIMLESS*/*TIM*, *RORA*, *RORB*, *RORC*, *NPAS2*, *NR1D1*, *NR1D2*, and *CSNK1E/CKIε* (Table 1) [12, 13]. Some of these genes constitute transcription-translation feedback loops. Basic helix-loop-helix heterodimeric transcription factors (*CLOCK*/*BMAL1* and *BMAL1*/*NPAS2*) regulate gene expression in the negative feedback way (*CRY1*, *CRY2*, *PER1*, *PER2*, and *PER3*) [14]. A mouse model with mutation of a *PER2* phosphodegron showed a longer circadian period in behavioral analysis. Simultaneously, nuclear protein expression of PER1, CRY1, and CRY2 was also increased, probably due to stabilization of PER2-containing complexes [15].

Circadian rhythms govern the development and behaviour of individuals [16, 17], in which disruption is associated with various diseases, including cardiac diseases, neuronal diseases, and cancers [18–20]. The circadian genes were differentially expressed and were involved in the initiation and progression of various cancers including HCC [21]. In addition, these circadian genes also have prognostic and therapeutic potential [21].

## 3. The Regulation of Circadian Genes in HCC

### 3.1. The Genetic Variation of Circadian Genes and HCC

Defects in circadian genes are closely associated with increased risk of cancer [22, 23]. The susceptibility and prognosis of cancer patients are significantly related to the genetic variation of circadian rhythm genes. Transcatheter arterial chemoembolization (TACE), one of the first-line forms of treatment for unresectable HCC, notably improves the recurrence-free survival (RFS) and overall survival of patients [24, 25]. In a cohort of 448 Chinese patients with unresectable HCC treated by TACE, two single-nucleotide polymorphisms (SNPs) (rs1053096 and rs2305160) were identified in the *NPAS2* gene, which showed significant associations with increased death risk of HCC patients [26]. Moreover, the occurrence of rs1053096 was related to increased expression of *NPAS2*, which may be a pivotal element that affects the prognosis of patients [26]. The other SNP rs2305160 was also related to the risk of breast cancer, prostate cancer, lymphocytic leukemia, and non-Hodgkin's lymphoma [27–30]. However, the clinical outcome varies significantly among HCC patients. Effective and specific biomarkers are still needed in predicting the responses of patients after TACE treatment.

Zhao et al. proposed that SNPs of circadian genes serve as potential prognostic biomarkers. In the study, effects of 12 functional SNPs from 5 circadian genes (*CRY1*, *CRY2*, *PER1*, *PER2*, and *PER3*) were assessed in a cohort of 337 unresectable Chinese HCC cases. It was found that a functional SNP in *PER3* gene (rs2640908) was significantly related to the overall survival rate of HCC patients treated with TACE. The SNP rs2640908 was predominant in late-stage HCC patients, particularly old-aged those with large tumor size, increased serum *α*-fetoprotein, and advanced TNM stage [31]. They further evaluated 13 functional SNPs from the same 5 circadian genes in another cohort of 489 Chinese HCC patients who underwent radical resection. SNPs in *PER1* (rs3027178), *PER3* (rs228669 and rs2640908), and *CRY1* (rs3809236) were significantly linked to overall survival rate, while SNPs in *PER1* (rs3027178), *PER3* (rs228729), and *CRY1* (rs3809236) were significantly related to RFS of HCC patients. Besides, the wild genotype of rs228729 in *PER3* is also a risk factor which contributes to the RFS in HCC patients [32]. These findings indicate that SNPs in circadian genes may act as independent biomarkers of prognosis for HCC patients (Table 2). However, more samples from different ethnic populations are still needed to confirm this result. In addition, a patient's genetic background should be considered in optimizing HCC treatment.

The potential mechanism of some SNPs in circadian genes was predicted using bioinformatics method [32]. The SNPs were located at the transcription factor binding region (rs228729 in *PER3*, rs3809236 in *CRY1*) and exonic splicing enhancer region (rs3027178 in *PER1*, rs228669 and rs2640908 in *PER3*), which affect the expression and sequence of mRNAs, respectively [32]. In addition, dozens of SNPs in circadian genes have been identified in endocrine cancers, including breast, ovarian, pancreatic, and prostate cancer, which have been summarized by Morales-Santana et al. [13]. Whether these SNPs are related to the risk of HCC is still unknown. Although SNPs may have biological functions affecting the prognosis of HCC, the underlying mechanisms governing the association between SNPs and the risk of HCC remain unclear. Further experiments are needed to identify other SNPs that may have diagnosis and prognosis potential on HCC.

### 3.2. The Epigenetic Modification of Circadian Rhythms in HCC

The alteration of gene expression in HCC was commonly caused by epigenetic modification occurring in DNA, RNA, and histone [33–35]. These epigenetic changes are also potential prognostic markers for HCC [34].

#### 3.2.1. DNA Modification

DNA methylation serves as a major epigenetic DNA modification in HCC, which also possesses diagnostic and prognostic potential [36]. In HCC cells, promoter methylation was found in both *PER1* and *CRY1* but not in *PER2*, *PER3*, *CRY2*, and *TIM* [11]. The promoter methylation in *PER1* and *CRY1* was also observed in endometrial cancer [37]. Therefore, the promoter methylation in circadian genes may play important roles in cancer.

#### 3.2.2. RNA Modification

N6-Methyladenosine (m6A) methylation is the most abundant posttranscriptional RNA modification. m6A methylase METTLs, demethylase ALKBHs, and YTHDF family proteins are pivotal participants of m6A methylation. m6A RNA methylation is involved in circadian rhythm regulation. m6A sites were found in the transcripts from *Per1*, *Per2*, *Per3*, *Clock*, *Nr1d1*, and *Nr1d2* [38–40]. METTL16 and ALKBH15 are important enzymes involved in the process of m6A. The mutation of METTL16 and ALKBH15 was associated with poor overall survival and RFS of HCC patients [41]. The expression of *ALKBH5* was downregulated in HCC tissues, which is associated with poor survival of HCC patients. *ALKBH5* could inhibit the proliferation and invasion of HCC cells both in vivo and in vitro [42]. *ALKBH5* was positively correlated with *PER1* expression in pancreatic cancer tissues and could prevent pancreatic cancer progression by increasing *PER1* mRNA expression in a m6A-dependent manner [43]. This mechanism may be also involved in HCC. m6A-related factors METTL3, YTHDF2, and ZC3H13 were associated with poor prognosis of HCC patients [44]. Knockdown of Mettl3 could induce circadian period elongation and RNA processing delay. When m6A methylation is inhibited, the uncoupling of nucleocytoplasmic distribution was observed between Per2 and Arntl [40]. However, the role and related regulatory mechanism of m6A methylation on circadian rhythms in HCC remain largely unelucidated.

#### 3.2.3. Histone Modification

Histone methylation and acetylation are vital for oscillation of circadian genes. *MLL3* was a frequently mutated gene related to the pathogenesis of HCC [45]. The histone methyltransferase encoded by *MLL3* could modulate more than a hundred of circadian “output” genes in the liver. Inactivation of MLL3 also compromises the oscillation of circadian gene promoters, including *Bmal1*, *Cry1*, and *Per2* [46]. Histone acetyltransferase p300 was highly expressed in HCC tissues and correlated with the malignancy of HCC. The inhibition of p300 by a specific inhibitor C646 inhibited invasion of HCC cells (Huh7, HLE, and SK-HEP1) [47]. Etchegaray et al. found that p300 precipitates with CLOCK/Bmal1-mediated transcription of *Cry1*, *Per1*, and *Per2* by regulating histone H3 acetylation at the promoters of these genes [48]. Doi et al. further showed that CLOCK is a DNA binding protein that possesses histone acetyltransferase activity, which can be enhanced by BMAL1 [49]. CLOCK is also involved in acetylating of BMAL1. Acetylated BMAL1 recruits CRY1 to the CLOCK-BMAL1 complex and represses transcription [50].

Histone deacetylase is also an important regulator of circadian rhythms, with an opposite role of histone acetylase. Valproic acid and trichostatin A, two histone deacetylase inhibitors, were found to increase H3 acetylation and regulate Per2 oscillations in an in vitro study [51]. In a mouse model, histone deacetylase 3 is activated by Ncor1 and thus involved in the inhibition of Bmal1 expression [52]. SIRT1 is an NAD(+)-dependent protein deacetylase, which was upregulated in HCC [53]. SIRT1 could bind CLOCK-BMAL1 in a circadian manner and promote PER2 deacetylation, thus regulating circadian gene expression, including Bmal1, Cry1, and Per2 [54].

These findings indicated that altered epigenetic control of circadian gene expression plays a substantial role in HCC progression. More studies are still needed to depict the coherent picture of the regulatory system.

### 3.3. The Transcriptional Regulation of Circadian Genes

The liver has the largest proportion of rhythmically expressed genes than other organs [55]. A series of researches were conducted to uncover the potential transcriptional regulation mechanism of circadian genes. The circadian machinery is driven by transcription regulators. The expression of CRY1 is lower in HCC tissues and cell lines. Overexpression of CRY1 inhibited the proliferation and promoted apoptosis of HCC cells [56]. The upregulation of transcription factor SREBP1c promoted HCC progression and metastasis, which may be related to the enhancement of CRY1 expression by targeting the sterol regulatory element and E-BOX motif in the promotor of CRY1 [57, 58]. Liu et al. constructed a transcription factor-based regulatory network in HCC using bioinformatics methods. They found that EGR1, FOS, and FOSB are differentially expressed transcription factors, which are key genes in the regulatory work [59]. EGR1 is highly expressed in HCC tissues, which could promote the proliferation of HCC cells and has prognostic implication in HCC [60–63]. The regulatory effect of EGR1 on BMAL1 has been identified in the SCN using the EGR1-deficient mice [64]. This regulatory mechanism may also exist in HCC.

Recently, Simak et al. revealed novel circadian transcriptional regulators in mammals using the Boolean function network (BFN) method and validated the results by previous high-throughput studies. They identified 93 and 95 transcriptional circadian regulators in mouse and rat livers, respectively. Some of the identified transcriptional regulators were shown to be associated with HCC, including Esr1, Smad4, Ctnnb1, Eno1, Gmnn, Trim24, Dnmt3b, Irf2, Rb1, Nfkbia, and Apex1 [65]. For example, Esr 1 is lower expressed in HCC cells. The expression of Esr1 is negatively related to the proliferation, migration, and invasion of HCC cells [66]. The activation of Smad4 is associated with metastasis and poor prognosis of HCC [67, 68]. Eno1 could upregulate integrin *α*6*β*4 expression thus promoting the growth and metastasis of HCC [69]. The involvement of these transcription factors may partially be through the regulation of circadian genes. Therefore, these circadian regulators have application potential in molecular medicine for HCC. Further molecular biology experiments are needed to validate the regulation of these transcription factors on circadian genes and its potential for HCC treatment.

### 3.4. The Posttranscriptional Regulation of Circadian Genes by Noncoding RNAs

Noncoding RNAs are emerging as important regulators of circadian rhythms and thus involved in the pathogenesis of HCC. Depending on the length and structure, noncoding RNAs could be classified as microRNAs (miRNAs), long noncoding RNAs (lncRNAs), and circular RNAs (circRNA) [70].

miRNAs are generally implicated in the regulation of gene expression at post-transcriptional level. Inhibition of miR-34a (an onco-miRNA) decreased proliferation and invasion of cholangiocarcinoma cells, which are related to the upregulation of *Per1* [71]. Aberrant expression of miR-34a is also involved in the initiation and progression of HCC [72–74]. However, whether the effect of miR-34a is through the regulation of circadian genes in HCC needs to be clarified. In addition, miR-10a was markedly upregulated by hepatitis C virus (HCV) infection, and overexpression of miR-10a impairs liver metabolism through the inhibition of Bmal1 in cirrhosis with HCV infection [75].

**Figure 1:**
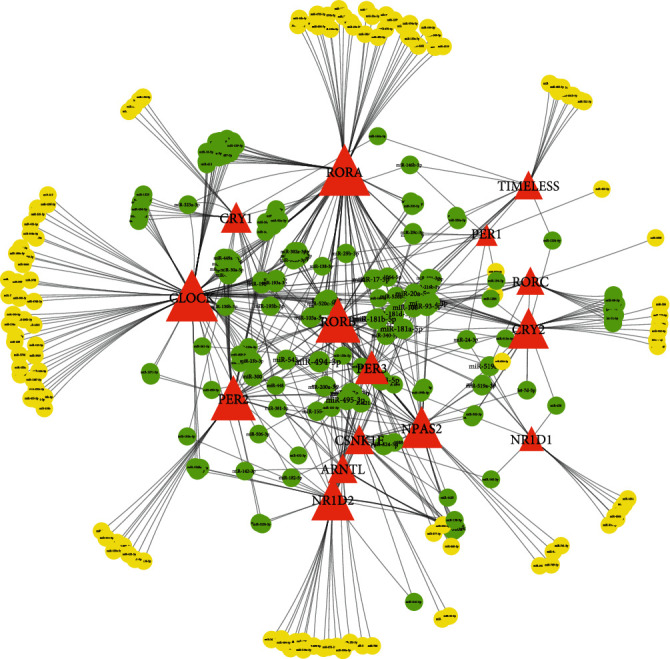
The predicted coregulation networks of circadian genes and microRNAs. The network contains 292 nodes (including 15 mRNAs and 277 miRNAs) and 586 edges. The red triangle nodes represent circadian genes. The circular nodes represent microRNAs (yellow nodes: connect one circadian gene; blue nodes: connected two or more circadian genes).

Polo et al. established a network using dysregulated genes in HepG2 cells and found that *CLOCK* was associated with the hub nodes of the network through CKAP5. Simultaneously, CKAP5 was associated with three circadian-related genes (CSNK1E, CSNK1D, and HDAC4). Furthermore, it was found that miR-195-5p, miR-192-5p, miR-122-5p, and miR-101-3p were involved in the dysregulation of circadian genes which led to HCC [76]. A series of miRNAs targeting circadian genes have been identified using ENCORI (http://starbase.sysu.edu.cn/) [77]. miRNAs identified by three or more prediction software were visualized (Figure 1) using Cytoscape (3.7.2) [78]. The function of some of these miRNAs (miR-494-3p, miR-21-5p, miR-30e-5p, miR-200c-3p, etc.) has been reported in HCC [79–82]. Circadian genes sharing the same miRNA may have more intimate interactions that require further investigation. On the other hand, circadian genes and miRNAs also have a feedback loop. In the liver of Clock *Δ*19 mutant mice, a total of 61 and 57 miRNAs were differentially expressed at zeitgeber time (ZT) 2 and 14, respectively, which were mainly involved in “pathways in cancer,” “PI3K-Akt signaling pathway,” and “MAPK signaling pathway.” Among these miRNAs, miR-340-3p (targeting Clock, Per1, and Cry2), miR-669d (targeting Per2), miR-374 (targeting Per3), and miR-338-5p (targeting Nr1d1) directly targeted core circadian genes [83]. These indicate that the interaction between miRNAs and circadian genes may play a vital role in HCC.

lncRNAs are considered as novel prognostic biomarkers in patients with HCC [84, 85]. However, investigations focusing on the function and mechanism of lncRNAs on the regulation of circadian genes are still limited. lncRNA highly upregulated in liver cancer (HULC) is prominently expressed in clinical HCC tissues [86]. The upregulation of HULC by hepatitis B virus (HBV) X protein promotes proliferation of LO2 and HepG2 cells [87]. Furthermore, HULC was positively correlated with CLOCK, and the overexpression of HULC upregulated the expression of CLOCK and its downstream circadian oscillators by targeting the 5′UTR of CLOCK mRNA. In addition, overexpression of HULC promoted the growth of HCC cells both in vitro and in vivo, which is related to the regulation of CLOCK [88]. lncRNAs may modulate hepatocarcinogenesis through disruption of the circadian rhythm.

circRNA is a type of regulatory noncoding RNA with a circular structure. A total of 527 circRNAs were identified to be differentially expressed in HCC tissues, with 174 upregulated and 353 downregulated [89]. Using bioinformatics, a functional circRNA-miRNA-mRNA regulatory network has been established, which may promote the identification of molecular biomarkers and therapeutic targets for HCC [90]. A database, CirGRDB (http://cirgrdb.biols.ac.cn), provided the genome-wide deciphering circadian genes and regulators, which will provide valuable insights into the investigation of circadian-related diseases [91]. These findings provide new insights into the involvement of noncoding RNA regulatory mechanisms on circadian rhythm involved in hepatocarcinogenesis.

## 4. The Function and Mechanisms of Circadian Genes in HCC

### 4.1. The Disruption of Circadian Rhythms as a Risk Factor for HCC

Chronic infection with hepatitis virus, exposure to carcinogens (e.g., aflatoxin-contaminated foodstuffs), and diabetes are all risk factors for HCC [92–95]. These risk factors are also related to the disruption of circadian rhythms.

HBV and HCV infection are closely related with end-stage liver diseases, such as liver cirrhosis and HCC [96–98]. It was found that the circadian clock index was lower in HBV-infected HCC tissues than normal tissues, which indicates HBV might contribute to the disruption of circadian rhythms in HCC [99]. Hepatitis B-X (HBx) protein encoded by HBV genome plays crucial roles not only in replication of HBV but also in the process of HBV-induced hepatocarcinogenesis [100]. In an artificial modified cell line, Bel-7404-HBx cells (a stable HBx-expressing cell line), the mRNA expression level of the *Clock*, *Per1*, and *Per2* genes was higher, while *BMAL1*, *Per3*, *Cry1*, *Cry2*, and *CKIε* was lower compared with that in Bel-7404 cells. This implies that HBx distorts circadian clock gene expression and could be involved in the development of HCC [101]. Benegiamo et al. examined the interplay between HCV infection and the expression of circadian genes using two cellular models (Huh-7 and OR6 cell lines) [102]. They found that the HCV genotype 1b, while not genotype 3a, induced profound alterations in circadian genes, as manifested by the downregulation of *PER2* and *CRY2* expression. Overexpression of *PER2* resulted in a significant reduction in HCV RNA replicating levels and restoration of the disrupted expression pattern of a subset of interferon-stimulated genes in OR6 cells (with HCV genotype 1b). In addition, *PER2* was markedly localized to the nucleus in liver biopsies taken from HCV patients with genotype 1b infection, which is coherent with an autoinhibitory transcriptional feedback loop [102].

Filipski et al. documented the characteristics of liver carcinogenesis following chronic exposure of mice to a cancer initiator diethylnitrosamine (DEN). DEN significantly interrupted the circadian rhythms in rest activity, which also affected the body temperature in all the mice. From the study, it was also discovered that chronic jet lag reduced rest activity and body temperature rhythms and increased growth of tumors induced by DEN in mice, which was at least partially related to the downregulation of p53 and upregulation of c-Myc. These findings indicate that the disruption of circadian rhythms may facilitate the initiation and progression of liver cancer [103]. Therefore, circadian coordination may be crucial in curtailing and/or reversing cancer development after exposure to carcinogens.

Several studies have revealed the relation between diabetes and the disruption of liver circadian rhythm. Daily patter of Per2 was disrupted in the liver of streptozotocin (STZ)-induced diabetic rat [104]. Similar phenomenon was also observed in mice, and administration of insulin recovered the rhythm of Per2 [105]. Hofmann et al. investigated the effect of type 1 diabetes on the rhythmic expression of circadian genes. The expression pattern alteration of circadian genes was observed in the liver of spontaneous (LEW.1AR1-iddm rat) and STZ-induced diabetic rat. *Per1* and *Bmal1* mRNA showed basically antiphasic diurnal oscillation expression patterns. There expression levels in certain time point were affected by diabetes [106].

The above findings suggest that the alteration of circadian genes could be induced by various HCC inductors. The disruption of circadian rhythms may be a risk factor for HCC.

### 4.2. The Clinical Relevance of Circadian Genes in HCC

Some studies have proved that circadian genes are abnormally expressed in substantial malignant tumors, and their expression levels are strongly linked to the degree of malignancy and prognosis of several tumors [107, 108]. A disruption in the circadian gene expression is also a common characteristic of HCC and associated with clinical manifestations observed in HCC patients [109].

Lin et al. detected the level of circadian genes (*PER1*, *PER2*, *PER3*, *CRY1*, *CRY2*, *CLOCK*, *BMAL1*, *CKIε*, and *TIM*) expression in 46 HCC and paired noncancerous tissues for the first time. The expression of *PER1*, *PER2*, *PER3*, *CRY2*, and *TIM* was reduced in HCC tissues, with no significant differences of *CLOCK*, *BMAL1*, *CRY1*, and *CKIε*. However, *PER2* and *PER3* negatively correlate with tumor size, and *TIM* negatively correlates with tumor grade [110]. Similarly, Yang et al. also found that the *PER1*, *PER2*, *PER3*, and *CRY2* mRNA expressions were significantly decreased in HCC tissues compared to paired noncancerous tissues, while no significant difference was observed in *CLOCK*, *BMAL1*, *CRY1*, and *CK1ɛ* [101].

Li et al. reported that *CLOCK* expression was increased in HCC tissues than in the adjacent nontumor liver tissues, which is correlated with tumor size, TNM stage, and portal vein invasion. HCC patients with lower *CLOCK* expression level had a longer rate of overall survival period and RFS time than those with high *CLOCK* expression. Furthermore, knockdown of *CLOCK* significantly inhibited the proliferation of HepG2 cells [109]. These reports show the involvement of circadian genes in HCC progression.

Additional oncogenes/tumor suppressor genes related to circadian genes/clock-controlled gene pairs have also been identified by Salavaty et al.; they include *CCNE1*/*SREBF1*, *SMO*/*LEF1*, *KRAS*/*CDK1*, *RTKN*/*TCF3*, *BCL6*/*WEE1*, *WRN*/*CSNK1D*, and *SMAD3*/*WEE1* [111]. Among these genes, *CCNE1*, *SMAD3*, *BCL6*, and *KRAS* have been confirmed to be clock-controlled [112–115]. Using RNA sequencing data from TCGA database, Qiu et al. analyzed the relation between circadian genes and HCC and found that the expressions of *CRY2* and *RORA* were positively correlated, while *NPAS2* and *TIM* were negatively correlated with overall survival of HCC patients. *CRY2*, *RORA*, *NPAS2*, and *TIM* were highly expressed in well-differentiated groups (G1 and G2) compared with poorly differentiated groups (G3 and G4). Besides, *RORA* and *NPAS2* were also positively associated with age of HCC patients [116]. These findings indicated that the expression of circadian genes was altered in HCC, which are potential biomarkers for HCC.

### 4.3. The Effect of Circadian Genes on the Progression of HCC

Studies have shown that circadian genes such as *Per1*, *Per2*, and *Per3* were all downregulated in HCC [101, 117, 118]. Sato et al. reported that the knockdown of *PER1* inhibited the proliferation of HepG2 cells, which was accompanied by the increase in the expression of cleaved PARP, cleaved Caspase-7, cleaved Caspase-9, and p53; the expression levels of Bax, Bcl-2, Bid, and c-Myc proteins remained unchanged, while cleaved Caspase-3 was not detected [117]. Mteyrek et al. examined the role of *Per2* using transgenic mice with *Per2* loss of function mutation (Per2^m/m^). It was discovered that *Per2* mutation disrupted the circadian rhythm and altered the expression levels of genes related to proliferation (c-Myc and Ccnb1), genomic instability (ATM, Wee1, and Ccnb1), and inflammation (IL-6 and TNF-*α*). These changes made Per2^m/m^ mice more likely to develop liver cancer after exposure to DEN, indicating that circadian gene *Per2* acts as a tumor suppressor in the liver [118].

The effects and mechanism of other circadian genes in HCC have also been discovered one after another. *BMAL2* is reported to be downregulated in HCC tissues [119]. Overexpression of antisense BMAL2 RNA reduced cell cycle time and decreased Caspase-3 activity, thus enhancing the proliferation of 293EBNA cells [119]. Elgohary et al. demonstrated that the gene and protein expression levels of TIM were upregulated in HCC samples from human tissues compared with that in nontumor liver tissues. Consistently, the elevated expression level of TIM was detected in Hep3B, HepG2, HuH6, HuH7, and PLC/PRF/5 cells. The knockdown of TIM inhibited the viability, caused cell cycle G2 arrest, and induced apoptosis in HepG2 and Hep3B cells [120]. Reduced migration ability was also observed in Hep3B cells after TIM knockdown. The effect of TIM downregulation was mediated via the phosphorylation of checkpoint kinase 2 (CHEK2) and the reduced expression of eukaryotic elongation factor 1A2 (EEF1A2) [120], which is an oncogene that positively correlated with the survival of HCC patients [121, 122]. Subsequently, Zhang et al. also confirmed that TIM is highly expressed in HCC cell lines (SK-HEP-1, SMMC-7721, MHCC97L, MHCC97H, and HepG2) compared with that in normal hepatic cell line HL-7702. The upregulation of TIM promoted the proliferation of HCC cells by reprogramming glucose metabolism, presented as enhanced production of lactic acid, by inhibiting p53 expression [123].

Yuan et al. observed that NPAS2 was frequently upregulated in HCC compared to paired adjacent nontumor tissues, which was associated with tumor progression and worse prognosis. NPAS2 promotes the growth of HCC cells in the in vitro and in vivo models, manifested by an enhanced proliferation of cells and inhibition of cell death [124]. The effect of NPAS2 was primarily mediated by upregulation of CDC25A phosphatase transcription, followed by dephosphorylation of CDK2/4/6 and Bcl-2. Furthermore, NPAS2 heterodimerizes with BMAL1 which directly binds to the promoter of CDC25A, indicating an important role of NPAS2/BMAL1 heterodimer in CDC25A transactivation mediated by NPAS2 [124]. CDC25A is highly expressed in HCC and is associated with poor survival [125]. Circadian genes may have a synergistic effect with each other and might influence the occurrence of HCC. Further studies are still needed to fully understand the molecular mechanisms of circadian genes in HCC pathogenesis. In addition, a recent study showed that *NR1D2* was highly expressed in HCC tissues and related to poor overall survival of patients, which may accelerate HCC progression by promoting epithelial-to-mesenchymal transition [126].

## 5. Circadian Gene-Based Cancer Intervention Strategies

Some medications have regulatory effects on circadian rhythms during cancer treatment. The nuclear hormone receptors REV-ERB*α* (encoded by NR1D1) and REV-ERB*β* (encoded by NR1D2) are components of the circadian rhythm. Sulli et al. found that two REV-ERB agonists, SR9009 and SR9011, are of high selectivity on a wide spectrum of tumors, with low toxicity [127]. Melatonin treatment could prompt the treatment of HCC [128, 129]. SR9009 inhibited the proliferation of Hep3B cells, which correlates with the upregulation of REV-ERB*α* and REV-ERB*β*, downregulation of BMAL1 and CLOCK, and Cyclin D1 and c-Myc protein [130]. Liver expression of Bmal1, Clock, Npas2, Ror*α*, and Sirt1 increased, whereas Cry1, Per1, Per2, Per3, CK1*ε*, Rev-erb*α*, and Rev-erb*β* decreased following DEN administration. Melatonin treatment prevented changes in the expression of circadian genes induced by DEN in mice. Furthermore, melatonin significantly potentiated the inhibitive effect of SR9009 on human Hep3B cells. The knockdown of BMAL1 promoted apoptosis, presented as increased expression of Bax, cleaved Caspase-3, and PARP1/2, and attenuated the proapoptotic and antiproliferative effect of melatonin in Hep3B cells [130]. Besides, Hou et al. observed the effect of electroacupuncture (EA) on the circadian rhythm on mice with HCC. EA regulated circadian rhythm of HCC mice and achieved the best efficacy at ZT 8 (15:00). EA at ZT 8 could restore the epigenetic gene expression in HCC models [131]. This may be due to a time-dependent change in circadian gene expression in HCC [132]. Therefore, restoration of circadian rhythms and abnormally expressed circadian genes might be a possible therapeutic target for the prevention and treatment of cancer.

Cisplatin is the front-line chemotherapeutic drug for HCC, which could exert anti-HCC effect by forming cisplatin-DNA adducts. The repair of cisplatin-DNA adduct was controlled by circadian genes (Npas2 and Dbp). These findings may help to improve optimal drug delivery regimen of cisplatin [133]. Immune therapy also plays a pivotal role in HCC treatment. The dominant effect of circadian clock on immune checkpoint pathway has been identified in sepsis [134]. The expression of BMAL1 is positively correlated with the clinical outcomes for melanoma patients with immune checkpoint inhibitors [135]. However, direct evidence on the influence of circadian genes on immunotherapy of HCC is still unknown. Further research findings about the correlation between circadian rhythm and cancer are expected to provide a theoretical basis for future time-based for drug delivery strategy.

## 6. Conclusions

**Figure 2:**
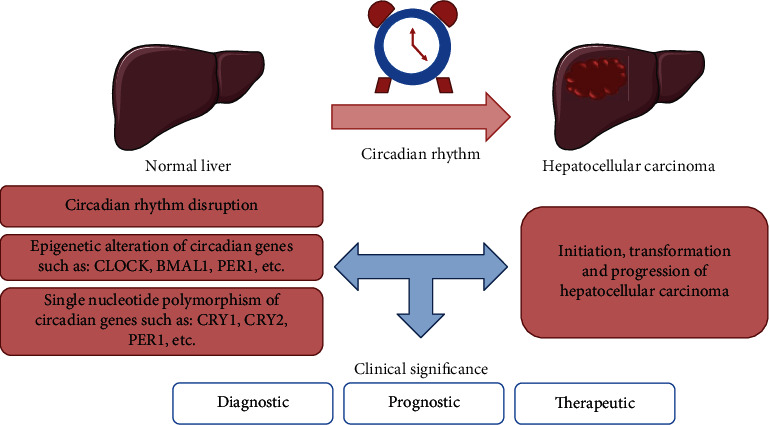
The role and clinical significance of circadian rhythms in hepatocellular carcinoma.

Circadian disruption has been involved in the development of various forms of human cancers. Studies have revealed that circadian genes play a pivotal role in hepatocarcinogenesis and progression, and their roles are summarized in Figure 2. Therefore, clarifying the effects and mechanisms of circadian rhythms on the organism has become increasingly important for the medical community. However, the exact molecular mechanisms of most circadian genes still require further exploration, especially the interaction between circadian genes and noncoding RNAs. A better understanding of the role of circadian rhythm would promote the discovery of novel biomarkers and therapeutic targets which will ultimately enhance the medication of HCC and other related diseases.

## Figures and Tables

**Table 1:** The current known mammalian circadian genes.

Circadian genes	Full name
PER1	Period-1
PER2	Period-2
PER3	Period-3
CLOCK	Circadian locomotor output cycles kaput
CRY1	Cryptochrome 1
CRY2	Cryptochrome 2
ARNTL/BMAL1	Arylhydrocarbon receptor nuclear translocator-like
TIM	Timeless
ROR	Retinoic acid-related orphan nuclear receptor
NPAS2	Neuronal PAS domain protein 2
NR1D1 and NR1D2	Nuclear receptor subfamily 1 group D members 1 and 2
CSNK1E	Casein kinase I epsilon

**Table 2:** The genetic alteration of circadian rhythm genes in hepatocellular carcinoma.

Circadian genes	SNP	Function of SNP	References
NPAS2	rs1053096	Associated with OS in transcatheter arterial chemoembolization-treated HCC patients	[26]
	rs2305160	Associated with OS in transcatheter arterial chemoembolization-treated HCC patients	
	rs9223		
	rs1562313		
	rs2305158		
	rs3811558		
CRY1	rs3809236	Associated with OS and RFS of HCC patients	[31, 32]
	rs1056560		
CRY2	rs6798		[31, 32]
	rs2292910		
PER1	rs3027178	Associated with OS and RFS of HCC patients	[31, 32]
	rs2585405		
PER2	rs934945		[31, 32]
	rs2304669		
PER3	rs228669	Associated with OS of HCC patients	[31, 32]
	rs2640908	Associated with OS of HCC patients	
	rs228729	Associated with RFS of HCC patients	
	rs172933		
	rs2859390		

HCC: hepatocellular carcinoma; OS: overall survival; RFS: recurrence-free survival.

## Abbreviations

CHEK2: Checkpoint kinase 2
circRNA: Circular RNA
DEN: Diethylnitrosamine
EA: Electroacupuncture
EEF1A2: Eukaryotic elongation factor 1A2
HBV: Hepatitis B virus
HBx: Hepatitis B-X
HCC: Hepatocellular carcinoma
HCV: Hepatitis C virus
lncRNA: Long noncoding RNA
m6A: N6-methyladenosine
miRNA: MicroRNA
Per2m/m: Per2 mutation
RFS: Recurrence-free survival
SCN: Suprachiasmatic nucleus
SNP: Single-nucleotide polymorphism
STZ: Streptozotocin
TACE: Transcatheter arterial chemoembolization
WHO: World Health Organization
ZT: Zeitgeber time
